# Insights From Quantitative Susceptibility Mapping: An Umbrella Review of Multiple Sclerosis

**DOI:** 10.1002/hsr2.72003

**Published:** 2026-03-16

**Authors:** Sadegh Ghaderi, Sana Mohammadi, Farzad Fatehi

**Affiliations:** ^1^ Neuromuscular Research Center, Department of Neurology, Shariati Hospital Tehran University of Medical Sciences Tehran Iran; ^2^ Department of Neuroscience and Addiction Studies School of Advanced Technologies in Medicine Tehran University of Medical Sciences Tehran Iran

**Keywords:** multiple sclerosis, neuroimaging, quantitative susceptibility mapping

## Abstract

**Background and Aims:**

Multiple sclerosis (MS) is a chronic inflammatory disease of the central nervous system characterized by demyelination and neurodegeneration. Conventional MRI techniques lack specificity for detecting subtle pathological changes, such as iron deposition and myelin dynamics. Quantitative susceptibility mapping (QSM), an advanced MRI method sensitive to tissue magnetic susceptibility (χ), has emerged as a promising tool for evaluating iron‐related pathology and demyelination in MS. This umbrella review synthesizes evidence from systematic reviews and meta‐analyses to consolidate the role of QSM in MS diagnosis, monitoring, and prognostication.

**Methods:**

Following PRISMA guidelines, a comprehensive search of PubMed, Scopus, Web of Science, and Embase identified seven studies (four meta‐analyses, three systematic reviews) encompassing 5389 MS patients, covering publications up to January 1, 2025. Methodological quality was assessed using AMSTAR 2, revealing moderate‐to‐low confidence in findings.

**Results:**

Key results demonstrated elevated χ in deep gray matter structures—particularly the globus pallidus, putamen, and caudate nucleus—in MS patients compared to healthy controls, correlating with clinical disability (e.g., Expanded Disability Status Scale scores) and cognitive dysfunction. Thalamic χ exhibited dynamic patterns, with reduced susceptibility in advanced disease stages. QSM outperformed conventional techniques (e.g., R2*, SWI) in detecting iron deposition, differentiating lesion types (e.g., paramagnetic rim lesions), and distinguishing MS from mimics. Rim‐enhancing lesions showed higher χ values linked to inflammatory activity, while white matter lesions reflected demyelination‐driven susceptibility changes. Despite QSM's diagnostic and prognostic potential, methodological heterogeneity and inconsistent standardization of PRL criteria limit comparability.

**Conclusion:**

This review underscores QSM's utility as a biomarker for MS pathophysiology while advocating for standardized protocols and longitudinal studies to validate its clinical translation.

## 1 Introduction

Multiple sclerosis (MS) is a chronic inflammatory disease of the central nervous system (CNS) characterized by focal areas of demyelination, axonal loss, and neurodegeneration, which reflect a wide range of neurological symptoms and often significant disability [1, 2]. Magnetic resonance imaging (MRI) plays a crucial role in the diagnosis, monitoring, and prognosis of MS [3]. However, conventional MRI techniques often lack specificity in detecting subtle pathological changes, particularly those related to iron deposition and myelin content [4].

The dysregulation of iron homeostasis in MS is increasingly recognized as a key component of its pathophysiology [5]. The underlying mechanisms are multifaceted, involving inflammation‐driven cellular damage that releases iron from damaged oligodendrocytes and myelin sheaths. This excess iron, which can be toxic, is sequestered by activated microglia and macrophages, particularly at the edges of chronic active lesions [6]. The clinical significance of this iron accumulation is profound; it catalyzes the production of reactive oxygen species, leading to oxidative stress, mitochondrial dysfunction, and further neurodegeneration, which, in turn, correlates with greater clinical disability and cognitive impairment [7]. Histopathological studies have provided direct validation for these imaging findings, confirming the presence of iron‐laden microglia/macrophages at the hyperintense rims of paramagnetic rim lesions (PRLs) seen on quantitative susceptibility mapping (QSM), establishing these rims as a marker of smoldering inflammation [8].

To address this limitation, QSM [9, 10] is an advanced MRI technique that is sensitive to the magnetic properties of tissues, referred to as maps of magnetic susceptibility (χ) [11], providing insights into the underlying “histological fingerprints” of MS [12]. The major sources of χ in the brain are paramagnetic iron, which is primarily stored in ferritin, hemosiderin, and diamagnetic myelin [13]. As such, QSM has shown promise in characterizing the heterogeneity of MS lesions, differentiating them from lesions associated with other conditions such as cerebral small vessel disease, and monitoring disease progression [14, 15, 16].

Unlike R2*, T2*, or susceptibility‐weighted imaging (SWI), which are indirectly sensitive to iron and prone to blooming artifacts, QSM provides an estimated measure of iron concentration, offering a more accurate and reliable assessment of iron‐related pathology [11, 17]. Beyond its sensitivity to iron, QSM also demonstrates the ability to detect myelin content, enabling the evaluation of the demyelination and remyelination processes. This capability arises from the diamagnetic properties of myelin, in contrast to the paramagnetic nature of iron. Consequently, QSM can facilitate a more comprehensive understanding of the complex interplay between iron deposition, demyelination, and inflammation in MS [18, 19, 20].

Thus, the primary aim of this umbrella review was to synthesize existing systematic reviews and meta‐analyses, consolidating the growing body of evidence from QSM studies on MS. By critically evaluating the literature, we aim to highlight the strengths and limitations of the current research, while identifying key areas for future exploration.

## 2 Methods

### 2.1 Search Strategy

To comprehensively synthesize existing evidence on QSM systematic reviews within the MS context, we employed an umbrella review methodology, which is a rigorous approach to evidence synthesis. This review adhered to the Preferred Reporting Items for Systematic Reviews and Meta‐analyses (PRISMA) guidelines [21]. The methodology itself drew upon established frameworks, specifically the Cochrane protocol for review overviews and the Joanna Briggs Institute approach for conducting umbrella reviews [22, 23]. Furthermore, we ensured methodological rigor by following the “Overviews of Reviews” methodology outlined by Cochrane Training (https://training.cochrane.org/handbook/current/chapter-v).

To identify the relevant literature, a comprehensive search strategy was developed and implemented across four electronic databases (PubMed, Scopus, Web of Science, and Embase). The search, conducted without language restrictions, covered publications up to January 1, 2025. This strategy utilizes Boolean operators (AND, OR) and a combination of relevant MeSH terms and keywords. Database‐specific search strategies are presented in Table S1.

### 2.2 Eligibility Criteria and Study Selection

Studies eligible for inclusion were systematic reviews or meta‐analyses of primary research evaluating QSM‐derived measures in the deep gray matter (DGM), such as χ, in adult participants diagnosed with MS according to established diagnostic criteria. Only reviews published as full‐text articles in peer‐reviewed journals were considered, with no restrictions on the publication date. Exclusion criteria included non‐systematic reviews (e.g., narrative and scoping reviews), original research articles, commentaries, editorials, quasi‐experimental studies, studies focusing on neuroimaging techniques other than QSM, pediatric populations, and non‐peer‐reviewed sources. The eligibility criteria were defined according to the Population, Exposure, Comparator, and Outcome (PECO) framework [24], specifying a population of individuals with MS, QSM as the primary exposure, healthy controls as the comparator, and neuroimaging findings as the outcome. Two independent reviewers (SG with 8 years of experience and SM with 4 years of experience) screened and selected the records using EndNote software. Discrepancies were resolved through discussion and consensus with a senior author (Professor of Neurology; CC). The screening process comprised title and abstract screening, followed by a full‐text review, adhering to PRISMA guidelines to ensure methodological rigor and transparency.

### 2.3 Data Extraction

Two independent reviewers (S.G. and S.M.) systematically extracted key data from the included systematic reviews and meta‐analyses to ensure a comprehensive and accurate synthesis of the findings. The extracted data were subsequently verified and synthesized by the primary reviewer (S.G.) to ensure consistency and reliability. The extracted data are presented in Table 1, which provides detailed information regarding the following study characteristics: study identification (author and year of publication), first author's country of origin, search period, databases searched, number and types of included primary studies, and number of patients. Table 1 also shows whether a meta‐analysis was conducted, the quality assessment tools employed (including evaluations for publication bias), and any additional statistical analyses performed. Table S2 reports the key findings pertaining to the QSM and related metric measurements.

**Table 1:** Main characteristics details for included studies.

Study, year	Continent	Search date (time frame or final search date)	Databases	Number of included QSM‐MS studies	Number of MS patients	Meta‐analysis	Guideline	Quality assessment/publication bias
Mohammadi et al. [25]	Asia	From inception to November 2023	PubMed, Scopus, and Web of Sciences	9	1074	Yes	PRISMA	Newcastle–Ottawa scale/Funnel plot and Egger test
Mohammadi et al. [26]	Asia	From inception to November 2023	PubMed, Scopus, and Web of Sciences	8	624	Yes	PRISMA	Newcastle–Ottawa scale/Funnel plot and Egger test
Voon et al. [27]	Europe	April 30, 2023	PubMed, Science Direct, Scopus, Web of Science, and Wiley Online Library	17	1903	Yes	PRISMA	The Cochrane Handbook's predefined quality assessment criteria
De Lury et al. [28]	North America	June 16, 2022	PubMed, Embase, and Web of Science	10	540	No	PRISMA	NR
Reeves et al. [29]	North America	December 5, 2022	PubMed and Embase	3	411	No	PRISMA	Modified criteria of the NIH Quality Assessment Tool for Observational Cohort and Cross‐Sectional Studies
Verma et al. [30]	Asia	NR	PubMed and Google Scholar	6	783	No	NR	NR
Gupta et al. [31]	North America	From inception to August 2016	Ovid MEDLINE, Ovid Embase, and the Cochrane	1	54	Yes	PRISMA	NA

Abbreviations: CIS, clinically isolated syndrome; MS, multiple sclerosis; NA, not applicable; NR, not reported; PMS, progressive multiple sclerosis; PPMS, primary progressive multiple sclerosis; PRISMA, Preferred Reporting Items for Systematic Reviews and Meta‐Analyses; QSM, quantitative susceptibility mapping; RRMS, relapsing remitting multiple sclerosis; SPMS, secondary progressive multiple sclerosis.

### 2.4 Assessment of Methodological Quality

Two reviewers (S.A. and S.M.) independently assessed the methodological quality of the included systematic reviews and meta‐analyses using the Assessment of Multiple Systematic Reviews 2 (AMSTAR 2) tool [32]. The AMSTAR 2 tool employs a four‐point response scale: “No,” “No Meta‐analysis,” “Partial Yes,” and “Yes”, which informs the overall confidence in the review findings. Based on these responses, the overall quality of each review was categorized into one of four levels: “High,” “Moderate,” “Low,” or “Critically low” confidence. This validated instrument comprised 16 items distributed across seven critical domains (Table S3).

### 2.5 Meta‐Analysis

Due to the heterogeneity in the reported quantitative findings, a meta‐analysis was not feasible within the scope of this umbrella review. Consequently, no further statistical analyses were performed. This review encompassed nine studies, including four meta‐analyses, each contributing valuable insights into QSM biomarkers in MS.

## 3 Results

### 3.1 Study Selection

**Figure 1:**
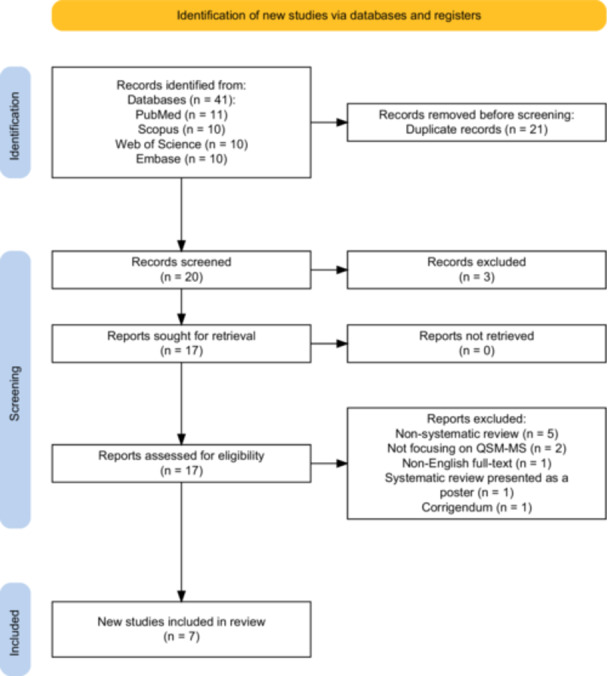
PRISMA flow diagram for systematic review for included studies. The diagram illustrates the different phases of study selection, from identification to final inclusion. The initial search across four databases (PubMed, Scopus, Web of Science, and Embase) identified 41 records. After the removal of 21 duplicates, 20 records were screened. From these, three records were excluded, and the remaining 17 reports were assessed for eligibility. During the eligibility assessment, reports were excluded for being non‐systematic reviews (*n* = 5), not focusing on QSM‐MS (*n* = 2), being a non‐English full‐text (*n* = 1), a poster (*n* = 1), or a corrigendum (*n* = 1). This resulted in a final selection of seven studies for inclusion in the review.

**Figure 2:**
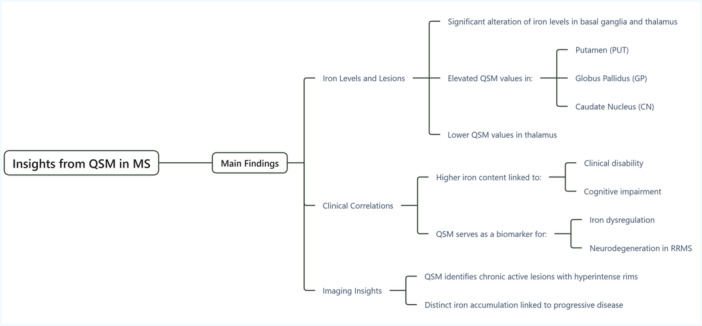
Concept map of the main findings from Quantitative Susceptibility Mapping (QSM) in Multiple Sclerosis (MS). The flow map summarizes the main findings of the review, which are categorized into three main branches: Iron Levels and Lesions, Clinical Correlations, and Imaging Insights. QSM reveals significant alteration of iron levels in the basal ganglia and thalamus, with elevated values in the putamen (PUT), globus pallidus (GP), and caudate nucleus (CN), and lower values in the thalamus. Clinically, higher iron content is correlated with greater clinical disability and cognitive impairment. QSM can also serve as a biomarker for iron dysregulation and neurodegeneration in RRMS. Key imaging insights include the ability of QSM to identify chronic active lesions with hyperintense rims and to detect distinct iron accumulation linked to progressive disease.

This umbrella review synthesized the findings from both meta‐analyses and systematic reviews. The study selection process (Figure 1). A comprehensive literature search, limited by publication date, yielded 41 records: 11 from PubMed, 10 from Scopus, 10 from the Web of Science, and 10 from Embase (Table S1). Following the removal of 21 duplicate records, the remaining 20 were subjected to title and abstract screening. Three records were subsequently excluded because they were outside the scope of this review. Based on their relevance to the research question, 17 papers proceeded to a full‐text review. Two systematic reviews, although broadly relevant, were excluded because of their lack of specific focus on MS and QSM [33, 34]. The reasons for the exclusion are shown in Figure 1. The final synthesis included seven studies [25, 26, 27, 28, 29, 30, 31]. Table 1 summarizes the characteristics of the included studies, and Table S2 details their main findings. This collective evidence provides insights into DGM iron content, lesion dynamics, clinical correlations, and the potential of QSM as a biomarker in MS (Figure 2).

### 3.2 Characteristics of Included Studies

Seven studies covering a broad range of geographic regions, databases, and study characteristics were included in this umbrella review. The number of publications is expected to range from 2017 to 2025. These employed various databases for literature retrieval, including PubMed, Scopus, Web of Science, Embase, Google Scholar, ScienceDirect, Wiley Online Library, Ovid MEDLINE, Ovid EMBASE, and the Cochrane Library. The included studies were conducted across multiple continents (Asia, Europe, and North America). These studies collectively incorporated data from 5389 patients with MS (only QSM‐MS studies reported in previous systematic reviews and meta‐analyses were included). The included studies comprised four meta‐analyses and three systematic reviews. Except for one study [30], all studies reported adherence to the PRISMA guidelines for the conduct and reporting of systematic reviews. However, the reporting of guidelines and methods for quality and publication bias assessments varied across the included studies. Quality assessment was conducted using several tools, including the Newcastle–Ottawa Scale [25, 26], modified criteria from the NIH Quality Assessment Tool for Observational Cohort and Cross‐Sectional Studies [29], and predefined quality assessment criteria from the Cochrane Handbook [27]. Publication bias was assessed using methods such as Egger's regression test and visual funnel plots [25, 26].

### 3.3 General Trends

The primary finding across all studies was the significant alteration of iron levels in various brain regions, particularly in the basal ganglia and thalamus, among patients with MS. Three studies consistently reported elevated QSM values in the putamen (PUT) (SMD: 0.37–0.40), globus pallidus (GP) (SMD: 0.48–0.60), and caudate nucleus (CN) (SMD: 0.40–0.54) in MS patients, especially those with relapsing‐remitting MS (RRMS), compared to HCs [25, 26, 27]. Notably, thalamic QSM values (SMD: −0.39) exhibit a contrasting trend [27], with a reduced χ observed in the later stages of MS [28].

### 3.4 Basal Ganglia‐QSM Findings

Several studies reported significant correlations between QSM findings and clinical disability [27, 28, 30]. Two studies observed that higher QSM values in the basal ganglia and thalamus PUT were associated with higher Expanded Disability Status Scale (EDSS) scores [27, 30]. Furthermore, as reported in the meta‐analysis by Voon et al. [27], rim lesions are associated with higher EDSS scores, and a positive association was found between normal‐appearing white matter (NAWM) QSM and EDSS.

Elevated iron content in regions such as the PUT, GP, and CN is closely associated with both clinical disability and cognitive dysfunction in patients [27, 28, 30]. These findings are particularly pronounced in secondary progressive MS (SPMS), where significant iron accumulation is linked to disease progression [28]. QSM measurements, consistent with R2* imaging, demonstrate that increased iron content in the PUT is a hallmark of MS, with elevated QSM values positively correlated with EDSS scores, indicating a relationship between higher iron levels and greater clinical disability [27, 30]. Furthermore, increases in PUT iron content strongly predict the Multiple Sclerosis Severity Score (MSSS), underscoring the role of iron deposition in disease severity [28].

Similarly, iron accumulation in the GP is a characteristic feature of MS, particularly SPMS, and is observed across various MS phenotypes, including RRMS and longitudinal MS [28]. The GP, along with other DGM regions such as the CN, exhibits greater iron deposition in patients with MS than in healthy controls (HC). This deposition correlates with increased EDSS scores, suggesting that higher iron levels in the GP contribute to greater disability [28]. Furthermore, iron accumulation in the GP is linked to MS‐related cognitive impairment [28], with QSM values showing a negative correlation with neuropsychological test performance, particularly in tasks involving recall and verbal fluency [27].

In CN, increased iron content was evident in both RRMS and SPMS patients, with longitudinal assessments showing an increase in CN iron levels from HC baseline to MS baseline [28]. Elevated CN iron levels are associated with a higher number of relapses, suggesting a connection with inflammatory activity during earlier disease stages. In contrast, studies on advanced MS phenotypes have reported reduced CN iron levels, indicating a shift in iron dynamics as the disease progresses. Greater CN iron deposition, along with iron in the PUT and GP, contributes to cognitive impairment in MS patients. In addition, increases in CN and PUT iron content strongly predict MSSS, highlighting the relevance of QSM as a biomarker for assessing disease severity and monitoring disease progression [28].

### 3.5 Thalamic‐QSM Findings

Thalamic iron levels have a complex relationship with disease progression in MS. While QSM reveals an overall lower χ in the thalamus compared to HC, there are variations in specific thalamic regions. For instance, QSM highlights regions with a more uniform appearance, whereas R2* imaging uncovers distinct regional iron clusters, such as those in the pulvinar nucleus of the thalamus, potentially indicating more focal areas of iron accumulation [28]. In terms of clinical outcomes, thalamic iron deposition correlates with EDSS scores, with greater iron deposition in the thalamus, along with other DGM regions such as the CN and GP, which are associated with greater disability in patients with MS [28]. The relationship between thalamic volume and QSM values is inconsistent, with some studies reporting a positive correlation and others reporting a negative correlation, suggesting a more complex interaction that may depend on disease stage or other factors [27]. In addition, thalamic iron levels may decrease in more advanced MS phenotypes, potentially owing to an inflammatory positive feedback loop [28]. This decrease in thalamic iron could represent a shift in disease progression, contributing to the observed clinical decline [27]. Interestingly, QSM also shows a negative correlation between thalamic χ and mobility, indicating that higher thalamic iron levels may be associated with worse motor function in patients [27]. This supports the idea that thalamic iron accumulation may play a role in the motor deficits observed in MS. However, despite these associations, many clinical measures showed mostly non‐significant relationships with thalamic QSM, indicating that the thalamus may not always serve as a direct predictor of clinical outcomes in MS.

### 3.6 QSM's Superiority

QSM has emerged as a promising non‐invasive imaging technique for both diagnostic and prognostic purposes. One primary study highlighted its potential as a non‐contrast alternative to gadolinium‐based imaging, which is particularly relevant given concerns regarding gadolinium retention [31]. QSM exhibits greater sensitivity to changes in iron deposition and demyelination than SWI, mitigating SWI's χ to aliasing and bias field artifacts [28]. QSM consistently outperforms other imaging modalities, such as R2* mapping and phase imaging, in detecting and characterizing MS lesions [28]. It also provides histopathological evidence supporting the superior sensitivity of QSM for detecting iron accumulation and demyelination [28]. In addition, one primary systematic review provided evidence regarding the high diagnostic accuracy (area under the curve = 0.95) of QSM in distinguishing between enhancing and non‐enhancing MS lesions [31].

### 3.7 QSM of MS Lesions: Paramagnetic Rims, Clinical Disability, and Lesion Evolution

Age and disease duration are critical factors that influence iron deposition patterns [25, 26]. One study highlighted increased basal ganglia QSM values in younger RRMS patients (< 40 years) [25], while another noted dynamic changes in white matter lesion (WML) QSM values over time [27]. Moreover, standardized criteria for PRL assessment, as suggested in another study [29], could further enhance QSM's diagnostic utility. There is little evidence of pronounced volume alterations in the thalamus and DGM [27, 28], with concurrent iron accumulation detected using QSM [27]. QSM measurements are also influenced by demyelination [28], and myelin loss is the main contributor to increased QSM values in the WMLs [27]. Disease stage also plays a significant role, with elevated iron levels observed in the early stages of RRMS and reduced thalamic χ in later stages [25].

Specifically, rim lesions showed higher QSM values than nodular‐enhancing lesions, reflecting an increase in the iron content at the lesion edges. The elevated χ in the rim region is driven primarily by iron deposition, which is also associated with inflammation. QSM signals in the rims of lesions correlate strongly with markers of inflammation and iron content, suggesting that iron accumulation plays a significant role in the pathophysiology of these lesions [27]. In the progression of MS, QSM values in lesions change over time [27, 30]. During gadolinium (Gd) enhancement, lesions exhibit lower QSM values, with a subsequent increase in QSM as lesions become non‐enhancing, peaking within the first 6 months post‐detection. However, this increase in QSM values began to decrease after approximately 2 years, although rim lesions may persist with elevated values for a longer period. This temporal pattern highlights the dynamic nature of iron deposition in MS lesions, particularly in the rims [27].

Rim lesions are also clinically relevant, as they have been associated with higher EDSS scores, indicating that these lesions contribute to greater disability in patients with MS. In addition, a positive association between NAWM QSM values and EDSS scores suggests that iron deposition in both lesions and surrounding tissue plays a role in disease severity [27]. MS lesions also exhibit a rapid increase in χ as they transition from enhanced to non‐enhanced stages, achieving a higher χ than NAWM [30]. Moreover, the detection of PRLs on QSM has proven reliable, especially when performed by experienced raters [29]. However, to improve consistency and facilitate comparisons across studies and clinical settings, standardized criteria and guidelines for PRL assessment are necessary [29]. Importantly, QSM distinguishes higher χ values in the rim + lesion region than in the rim‐lesion region, further supporting the notion that the rim may serve as a potential biomarker for MS [30].

### 3.8 Results of Methodological Quality Assessment

Based on the AMSTAR 2 guidelines (Table S2), we evaluated the methodological quality of the included systematic reviews, and the meta‐analyses were assessed using the AMSTAR 2 checklist (Table 2). This assessment revealed variability in methodological rigor across studies. Five studies [25, 26, 27, 29, 31] were classified as having a moderate methodological quality, while two [28, 30] were rated as low‐quality. Common weaknesses identified by the AMSTAR 2 assessment included the lack of an explicit list of excluded studies (Domain 7), inconsistent assessment of risk of bias in the primary studies (Domain 9), and failure to account for this risk of bias in the interpretation of the results (Domain 13). These findings highlight the need for greater adherence to methodological standards in future systematic reviews and meta‐analyses investigating QSM for MS.

**Table 2:** Results of the methodological quality assessment of included systematic reviews and meta‐analyses using the AMSTAR 2 Checklist (response options:

Study, year	Q1	Q2	Q3	Q4	Q5	Q6	Q7	Q8	Q9	Q10	Q11	Q12	Q13	Q14	Q15	Q16	Overall
Mohammadi et al. [25]	Y	PY	Y	PY	Y	Y	N	Y	Y	Y	Y	N	Y	Y	Y	Y	Moderate
Mohammadi et al. [26]	Y	PY	Y	PY	Y	Y	N	Y	Y	Y	Y	N	Y	Y	Y	Y	Moderate
Voon et al. [27]	Y	Y	Y	Y	Y	Y	N	Y	Y	Y	Y	N	Y	Y	Y	Y	Moderate
De Lury et al. [28]	Y	PY	Y	PY	Y	Y	N	Y	N	N	NM	NM	N	N	NM	N	Low
Reeves et al. [29]	Y	Y	Y	PY	Y	Y	N	Y	Y	Y	NM	NM	Y	Y	NM	Y	Moderate
Verma et al. [30]	Y	PY	Y	PY	N	N	N	Y	N	Y	NM	NM	N	N	NM	Y	Low
Gupta et al. [31]	Y	PY	Y	PY	Y	Y	N	Y	Y	N	Y	N	N	Y	Y	N	Moderate

Abbreviations: N, No; NM, No Meta‐analysis; PY, Probably Yes; Y, Yes.

## 4 Discussion

This review focuses on understanding the clinical and pathological implications of QSM findings in various aspects of MS, including DGM iron deposition, lesion characterization, longitudinal changes, and histological correlations. In line with previous studies [11, 35, 36, 37, 38, 39, 40, 41], MS pathology extends beyond WM involvement, as significant alterations are consistently observed in the DGM structures, particularly in the basal ganglia and thalamus. QSM measurements consistent with previous efforts revealed distinct patterns of iron dysregulation, with increased χ in the PUT, CN, and GP [11, 39, 42, 43, 44, 45, 46, 47, 48, 49, 50, 51], along with decreased χ in the thalamus [52, 53, 54]. These alterations may result from ongoing neurodegeneration, demyelination, and disruptions in iron metabolism, indicating their specificity for MS pathology [45, 55]. Notably, increased χ in PUT is associated with higher EDSS scores, reinforcing its potential as a biomarker for disease severity [26, 56]. Furthermore, the association between iron accumulation and cognitive dysfunction, particularly in recall and verbal fluency, the negative correlations between GP QSM values and neuropsychological test performance, and the predictive value of PUT and CN iron content for MSSS further emphasize QSM's clinical relevance of QSM as a potential surrogate marker for cognitive decline and as a biomarker for overall disease severity [57, 58]. However, the mechanisms that underlie these regional differences remain unclear. Although inflammation‐driven iron accumulation in MS lesions has been proposed, other studies suggest that iron deposition may result from demyelination and oligodendrocyte loss, necessitating further research to elucidate these complex interactions [59, 60].

Consistent with previous reviews [3, 33, 34, 55, 59, 61, 62, 63], this review highlights the role of QSM in detecting significant iron accumulation in DGM structures, such as the PUT, CN, and GP, for detecting and differentiating MS phenotypes [37], particularly in RRMS and SPMS, where the severity of iron deposition correlates with disease progression. Elevated QSM values in these regions are associated with greater clinical disability and cognitive impairment, underscoring their relevance for monitoring disease severity [28, 51, 64, 65]. Furthermore, longitudinal studies have emphasized the dynamic nature of iron deposition, with progressive increases observed in early MS stages and potential reductions in advanced disease phases [28, 30, 39, 66]. These findings, combined with evidence of thalamic atrophy and microstructural changes correlating with clinical outcomes [64, 67], reinforce the utility of QSM as a non‐invasive imaging tool for diagnosis, prognostication, and monitoring MS.

QSM has emerged as a more favorable and powerful imaging tool for characterizing MS lesions such as PRLs [68], particularly chronic active lesions, which exhibit distinctive iron accumulation patterns. Studies consistently highlight the presence of QSM rim‐positive (rim+) lesions, characterized by hyperintense rims indicative of iron‐laden macrophages and microglia, as a hallmark of persistent inflammation and ongoing demyelination [37, 69, 70, 71, 72]. These rim+ lesions are clinically significant, and their presence is associated with greater cortical thinning, thalamic volume loss, and more severe clinical disability, as reflected by higher EDSS scores [71]. Notably, sex differences were observed, with male MS patients exhibiting a significantly higher proportion of rim+ lesions than females, suggesting a potential influence of sex on lesion characteristics and disease progression [73].

QSM‐based longitudinal studies have provided critical insights into the dynamic nature of the evolution of MS lesions. In the early stages, newly formed lesions exhibit lower χ values, reflecting initial myelin breakdown [27]. Over time, these lesions demonstrated an increase in QSM values, peaking within 2 years, followed by a gradual decline as inflammation subsided and iron was redistributed. This temporal pattern reflects the interplay among inflammatory activity, myelin degradation, and iron metabolism [16, 74, 75]. In addition, baseline QSM measures in NAWM have been linked to biomarkers of neurodegeneration, such as higher serum neurofilament light chain concentrations and lower cerebrospinal fluid amyloid‐beta levels, suggesting that QSM may serve as a surrogate marker for early neuro‐axonal damage [76].

MRI, using post‐processing techniques, has long sought to capture the histological fingerprint of the brain non‐invasively, particularly focusing on iron and myelin, which play key roles in normal brain function and serve as significant markers in diseases involving neurodegeneration [12, 68, 72, 77, 78]. Histological studies have validated QSM findings by demonstrating iron accumulation in DGM structures and at the edges of rim lesions in post‐mortem MS brain tissue [27]. The integration of QSM with complementary MRI techniques, such as myelin water fraction (MWF) imaging, has enhanced our understanding of MS lesion development by confirming that increased QSM values in WM lesions are driven by both iron deposition and myelin loss [72, 79]. QSM also shows promise in characterizing the heterogeneity of MS lesions by distinguishing between different pathological subtypes [15]. Specifically, lesions with hyperintense QSM cores typically indicate myelin loss, whereas lesions with hypointense cores suggest iron accumulation.

This review highlights several advantages of QSM over other imaging modalities. QSM's increased sensitivity to changes in iron deposition and demyelination compared with SWI makes it a more robust tool for detecting and characterizing MS lesions [11, 28]. Its ability to mitigate SWI's χ to aliasing and bias field artifacts further enhances its reliability [28]. The reported high diagnostic accuracy of QSM in distinguishing enhancing from non‐enhancing MS lesions makes it a valuable tool for lesion characterization and monitoring of disease activity [27, 31]. Moreover, QSM's potential as a non‐contrast alternative to gadolinium‐based imaging is particularly relevant, given concerns about gadolinium retention [27, 80].

Despite significant advances in MS research enabled by QSM, several limitations and challenges remain. Our review is based on a relatively small number of included articles (seven), which reflects the specialized and emerging nature of synthesizing QSM research in MS through systematic reviews and meta‐analyses. Our scope was strictly limited to these types of reviews, not primary QSM studies, to maintain the umbrella review methodology. Furthermore, methodological variability across studies complicates direct comparisons and hinders the feasibility of a large‐scale meta‐analysis. This heterogeneity stems from several factors, including differences in MRI hardware (e.g., field strengths of 1.5T, 3T, or 7T), QSM acquisition parameters (e.g., sequence type, spatial resolution, echo times), and post‐processing pipelines. The latter includes varied approaches to background field removal (e.g., SHARP, V‐SHARP), dipole inversion algorithms (e.g., MEDI, STAR‐QSM), and the choice of a zero reference region, all of which can significantly influence the final susceptibility values and reduce comparability. Clinical validation of QSM as a reliable prognostic biomarker and tool for monitoring treatment response requires extensive longitudinal studies with larger cohorts. In addition, lesion heterogeneity (e.g., type and region) observed on QSM highlights the need for further research into the underlying biological mechanisms and their clinical significance. Combining QSM with other advanced MRI techniques, such as MWF imaging, diffusion tensor imaging, and magnetization transfer ratio, could provide a more comprehensive understanding of MS lesion pathology by simultaneously assessing demyelination, neuro‐axonal loss, and microstructural changes in both WM and GM [4, 37, 81]. Moreover, emerging techniques, such as intra‐voxel χ separation, have the potential to enhance the utility of QSM by independently quantifying paramagnetic (iron) and diamagnetic (myelin) components within lesions and the NAWM [1, 12, 82]. This approach could improve the differentiation of pathological processes underlying MS and refine the interpretation of χ signals. The validation of QSM findings through histopathological analyses is critical for establishing robust links between imaging biomarkers and tissue changes. Finally, QSM using ultra‐high‐field MRI enhances the detection and staging of MS lesions in the CNS [83].

## 5 Conclusions

QSM reliably detects iron accumulation in DGM structures, particularly the PUT, CN, and GP, with elevated susceptibility values strongly correlating with clinical disability and cognitive impairment. The technique's sensitivity to both paramagnetic iron and diamagnetic myelin enables the differentiation of MS lesions from other pathologies and provides insights into dynamic processes such as rim‐enhancing lesion evolution and demyelination. Notably, thalamic susceptibility patterns reflect disease progression, with reduced χ in advanced stages suggesting complex iron homeostasis shifts. QSM's superiority over conventional MRI techniques lies in its quantitative accuracy, reduced artifacts, and ability to disentangle iron from myelin effects, positioning it as a non‐invasive alternative to gadolinium‐based imaging.

## Author Contributions

**Sadegh Ghaderi:** conceptualization, investigation, writing – original draft, writing – review and editing, visualization, validation, methodology, software, formal analysis, project administration, data curation, supervision, resources. **Sana Mohammadi:** investigation, validation, visualization, writing – original draft, formal analysis, project administration, conceptualization. **Farzad Fatehi:** writing – review and editing, project administration, supervision, validation.

## Funding

The authors received no specific funding for this work.

## Ethics Statement

The authors have nothing to report.

## Conflicts of Interest

The authors declare no conflicts of interest.

## Transparency Statement

The lead author, Sadegh Ghaderi, affirms that this manuscript is an honest, accurate, and transparent account of the study being reported; that no important aspects of the study have been omitted; and that any discrepancies from the study as planned (and, if relevant, registered) have been explained.

## Supporting information


**Supplementary Table 1:** The search strategies used for database searches (01/01/2025). **Supplementary Table 2:** Main findings for included studies. **Supplementary Table 3:** AMSTRAR 2 checklist questions.

## Data Availability

The data that support the findings of this study are available from the corresponding author upon reasonable request.
