# Supplementary material for: Insights From Quantitative Susceptibility Mapping: An Umbrella Review of Multiple Sclerosis
Source: Health Sci Rep. 2026 Mar 16;9(3):e72003. doi: 10.1002/hsr2.72003 (PMC13097562; doi:10.1002/hsr2.72003)
Supplement: Supplementary file 1 — Supplementary Table 1: The search strategies used for database searches (01/01/2025). Supplementary Table 2: Main findings for included studies. Supplementary Table 3: AMSTRAR 2 checklist questions. [file HSR2-9-e72003-s001.docx]

| **Supplementary Table 1.** The search strategies used for database searches (01/01/2025) | | |
| --- | --- | --- |
|  | **Database** | **N** |
| PubMed | (("quantitative susceptibility Mapping"[All Fields] OR "QSM"[All Fields]) AND "multiple sclerosis"[MeSH Terms]) AND (meta-analysis[Filter] OR systematicreview[Filter]) | 11 |
| Scopus | TITLE-ABS-KEY ( "quantitative susceptibility Mapping" OR "QSM" ) AND TITLE-ABS-KEY ( "multiple sclerosis" ) AND TITLE-ABS-KEY ( "systematic review" ) OR TITLE-ABS-KEY ( "meta-analysis" ) | 10 |
| Web of Science (WOS) | TS=("quantitative susceptibility Mapping" OR "QSM") AND TS=("multiple sclerosis") AND (TS=("systematic review") OR TS=("meta-analysis")) | 10 |
| Embase | ('quantitative susceptibility mapping':ti,ab,kw OR qsm:ti,ab,kw) AND 'multiple sclerosis':ti,ab,kw AND ('meta analysis'/de OR 'systematic review'/de) | 10 |

| **Supplementary Table 2.** Main findings for included studies (AUC: area under the curve; CI: confidence interval; CN: caudate nucleus; DGM: deep gray matter; EDSS: Expanded Disability Status Scale; FLAIR: fluid-attenuated inversion recovery; GP: globus pallidus; HC: healthy controls; MSSS: Multiple Sclerosis Severity Score; NAWM: normal-appearing white matter; PRL: paramagnetic rim lesion; PUT: putamen; QSM: quantitative susceptibility mapping; RRMS: relapsing-remitting multiple sclerosis; SMD: standardized mean difference; SPMS: secondary progressive multiple sclerosis; SWI: susceptibility-weighted imaging; WML: white matter lesion; R2*: transverse relaxation rate.) | |
| --- | --- |
| Study, Year | Main Findings |
| Mohammadi et al. (2024)a ^1^ | - Meta-analysis (using Cohen's d^^[[1]](#footnote-1)^^) revealed significantly higher QSM values in the PUT (SMD 0.40, 95% CI 0.22 to 0.59), GP (SMD 0.60, 95% CI 0.50 to 0.70), and CN (SMD 0.40, 95% CI 0.15 to 0.66) of RRMS patients compared to healthy controls (HCs), indicating increased iron deposition in these basal ganglia regions. - No significant difference in overall thalamic QSM values was found between RRMS patients and HCs (SMD -0.33, 95% CI -0.67 to 0.01). - Younger RRMS patients (< 40 years) exhibited significantly higher QSM values in the PUT, GP, and CN compared to HCs, suggesting that iron accumulation in these regions may be more pronounced in earlier disease stages. - Thalamic QSM values were found to decrease in RRMS patients over 40 years of age. - Studies with a larger male population (> 25%) showed more significant differences in PUT and GP QSM values, suggesting a potential influence of sex on iron deposition patterns in these regions. - During the early stages of RRMS (disease duration < 9.6 years), QSM values were higher in the PUT, GP, and CN compared to HCs. Conversely, thalamic QSM values were lower in later stages of RRMS (disease duration > 9.6 years) compared to HCs. - QSM shows promise as a biomarker for understanding iron dysregulation and its potential role in neurodegeneration in RRMS, particularly within the basal ganglia (PUT, GP, and CN). - Further longitudinal research is needed to confirm these findings and investigate the complex relationship between iron deposition and disease progression in RRMS. |
| Mohammadi et al. (2024)b ^2^ | - Pooled meta-analysis (using Cohen's d) and individual studies consistently demonstrate significantly elevated putaminal iron levels in patients with RRMS compared to HC - A SMD of 0.37 (95% CI 0.177 to 0.58) quantifies this significant increase in putaminal iron in RRMS. - QSM is recognized as a promising technique for detecting and quantifying iron accumulation, a key pathological feature associated with RRMS. - Subgroup analyses confirm distinct patterns of putaminal iron alterations in RRMS compared to HC, supporting the growing evidence that iron accumulation plays a significant role in the pathophysiology of MS. - The observed increase in putaminal iron in RRMS aligns with existing research implicating iron in neuroinflammation and demyelination processes. |
| Voon et al. (2024) ^3^ | - DGM Susceptibility Changes: QSM reveals distinct alterations in DGM susceptibility in MS compared to healthy controls: - Increased Basal Ganglia Iron: Significant increases in QSM values (indicating higher iron content) are consistently observed in the basal ganglia: - Caudate: SMD (pooled) = 0.54 (95% CI: 0.39–0.70, I² = 46%), SMD (main) = 0.68 (95% CI: 0.25–1.11) - PUT: SMD (pooled) = 0.38 (95% CI: 0.19–0.57, I² = 59%), SMD (main) = 0.72 (95% CI: -0.03–1.47) (marginally significant) - GP: SMD (pooled) = 0.48 (95% CI: 0.28–0.67, I² = 60%), SMD (main) = 0.63 (95% CI: 0.14–1.13) - Thalamic QSM values are significantly reduced: SMD (pooled) = -0.39 (95% CI: -0.66–-0.12) (significant after outlier removal and in some subgroups), SMD (main) = -0.11 (95% CI: -0.70–0.48) - QSM values within WMLs hange over time: initial increase, followed by a plateau (around two years), and then a gradual decline. - Lesions show lower QSM values during Gd-enhancement. Rim-enhancing lesions exhibit higher QSM values than nodular-enhancing lesions. QSM values increase as lesions become non-enhancing, peaking within the first six months post-detection, then decreasing after approximately two years (later for rim lesions). - QSM lesion counts showed varying proportions of WMLs, cortical lesions, and mixed lesions. - Rim lesions and hyperintense QSM lesions were found. - PUT QSM values correlate positively with EDSS scores. - Rim lesions are also associated with higher EDSS scores and also found a positive association between NAWM QSM and EDSS. - Mostly non-significant associations were found between DGM susceptibility and clinical measures, with some exceptions: negative correlation between thalamic QSM and mobility; positive correlation between caudate QSM and Flanker's test performance; negative correlation between GP QSM and neuropsychological test performance (recall and verbal fluency). - Findings on the relationship between basal ganglia volume and QSM values are inconsistent (one study reported a negative correlation, while three reported a positive correlation between thalamic volume and QSM. - Iron content is the primary driver of QSM values in DGM and rim lesion edges. QSM signals in rims correlate with iron content and markers of inflammation. - Myelin loss is the main contributor to increased QSM values in WMLs. - Most studies found non-significant correlations between disease duration and DGM QSM values. - Sensitivity analyses (outlier removal, age-matching, 3T restriction, reference region exclusion) generally confirmed the main findings. Subgroup analysis for RRMS showed similar results with less heterogeneity in basal ganglia. |
| De Lury et al. (2023) ^4^ | - Postmortem studies demonstrated strong correlations between QSM, R2*, and histochemical detection of iron (Perls' Iron Stain), suggesting that ferric iron is the primary contributor to DGM susceptibility, rather than myelin or water content. - QSM exhibits greater sensitivity to changes in iron deposition and demyelination compared to susceptibility-weighted imaging (SWI), mitigating SWI's susceptibility to aliasing and bias field artifacts. - QSM is generally more reliable than R2* in detecting demyelination and DGM iron accumulation. - While QSM demonstrates greater sensitivity to MS-related demyelination and DGM iron changes, combining QSM with R2* can provide complementary information. - QSM tends to highlight structures with uniform appearance, whereas R2* reveals distinct regional iron clusters, potentially representing more focal areas of iron accumulation (e.g., in the pulvinar nucleus of the thalamus). - Both R2* and QSM measurements can be affected by brain atrophy, reducing their specificity for DGM iron. QSM measurements are also influenced by demyelination. - QSM-derived iron measurements have demonstrated superior sensitivity for detecting cognitive decline in MS compared to other imaging modalities like R2 and R2*, likely due to its enhanced sensitivity to both iron accumulation and demyelination. - Studies consistently report significant differences in DGM iron content between MS patients and healthy controls (HC) using QSM. - The GP and PUT show elevated iron levels in SPMS compared to HC, as measured by both QSM and R2*. - Increased iron in the GP is observed in comparisons of unspecified MS, RRMS, and longitudinal MS (including RRMS) to HC. - Studies have shown increased caudate iron content in comparisons of MS patients (including RRMS) to HC, and in longitudinal assessments from MS baseline to HC baseline. This increase in caudate iron has been correlated with a higher number of relapses, suggesting a link to inflammatory activity in earlier disease stages. Conversely, some studies including patients with advanced MS phenotypes have reported reduced caudate iron levels, indicating a potential shift in iron dynamics with disease progression. - Caudate iron content correlates with disability across all MS phenotypes. - QSM-detected increases in PUT and caudate iron content are strong predictors of the MSSS. - Greater DGM iron deposition, particularly in the caudate, thalamus, and GP, is associated with increased EDSS scores, indicating greater disability. - DGM iron deposition in the caudate, PUT, and GP contributes to MS-related cognitive impairment. - Thalamic iron levels may decrease in more advanced MS phenotypes, potentially due to an inflammatory positive feedback loop. - DGM iron accumulation is present even in the earliest stages of MS, although its relationship to phenotype conversion remains unclear. - Combining QSM and R2* using sparse classification can differentiate between iron accumulation and demyelination, showing greater specificity for iron deposition. - Iron deposition is suggested to be a downstream effect that exacerbates inflammation and oxidative damage in MS. |
| Reeves et al. (2023) ^5^ | - Misclassifications in QSM analysis could potentially be reduced by utilizing higher-resolution imaging (e.g., 7T), which would improve the delineation of paramagnetic vessels and the cortical ribbon. - Careful inspection of QSM/FLAIR co-registration accuracy is crucial to prevent misalignment of T2 lesion masks, which can impact QSM analysis. - PRL assessment on QSM can be reliable, especially when performed by experienced raters. However, standardized criteria and guidelines for PRL assessment are necessary to enhance consistency and enable comparisons across studies and clinical settings. - The rims of consensus PRLs were visible on phase imaging and across various QSM inversion algorithms, although with some differences in appearance. |
| Verma et al. (2022) ^6^ | - QSM is a more accurate and sensitive technique than phase imaging and R2* mapping for investigating MS, yielding higher magnetic susceptibility values in individuals with MS. - QSM can detect iron accumulation in MS lesions and DGM structures, providing insights into disease progression and severity. - QSM, in conjunction with lesion masks, facilitates the calculation and comparison of susceptibility values in different lesion groups categorized by image intensity or anatomical location. - MS lesions exhibit a rapid increase in magnetic susceptibility as they transition from enhanced to non-enhanced stages, achieving higher susceptibility than NAWM. - Longitudinal studies demonstrate an increase in lesion growth from enhanced to non-enhanced regions, reflected in quantitative susceptibility values. This confirms that QSM can track changes in lesion characteristics over time. - Iron deposition, reflected in susceptibility changes, is associated with MS, particularly in DGM regions. - MS patients exhibit lower susceptibility in the thalamus and higher susceptibility in the basal ganglia compared to HC. - QSM detects higher susceptibility values in the rim + lesion region compared to the rim - lesion region, suggesting this difference could serve as a biomarker for MS. - QSM values in the PUT have shown a positive correlation with the EDSS in MS patients, linking imaging findings to clinical disability. |
| Gupta et al. (2017) ^7^ | - Diagnostic Accuracy of QSM in Differentiating Enhancing and Non-Enhancing Lesions - high diagnostic accuracy for QSM in distinguishing enhancing from non-enhancing MS lesions, with an AUC of 0.95. - This high diagnostic accuracy is attributed to the ability of QSM to utilize specific relative susceptibility values. - The capacity of QSM to detect acute enhancing lesions aligns with observational data indicating an increase in lesion magnetic susceptibility as the lesion transitions from an enhancing to a non-enhancing state. - Non-contrast quantitative MRI techniques such as QSM offer the potential to reduce or replace the need for gadolinium-based contrast agents in the evaluation of MS lesions. - This reduction in gadolinium use could mitigate the risks associated with repeated gadolinium exposure and potentially contribute to a deeper understanding of MS lesion pathophysiology. |

| **Supplementary Table 3.** AMSTRAR 2 checklist questions | | |
| --- | --- | --- |
| Number | Quetion | Potential response |
| Q1 | Did the research questions and inclusion criteria for the review include the components of PICO? | - N: No - NM: No meta-analysis - PY: Probably yes - Y: Yes |
| Q2 | Did the report of the review contain an explicit statement that the review methods were established prior to the conduct of the review and did the report justify any significant deviations from the protocol? |  |
| Q3 | Did the review authors explain their selection of the study designs for inclusion in the review? |  |
| Q4 | Did the review authors use a comprehensive literature search strategy? |  |
| Q5 | Did the review authors perform study selection in duplicate? |  |
| Q6 | Did the review authors perform data extraction in duplicate? |  |
| Q7 | Did the review authors provide a list of excluded studies and justify the exclusions? |  |
| Q8 | Did the review authors describe the included studies in adequate detail? |  |
| Q9 | Did the review authors use a satisfactory technique for assessing the risk of bias [RoB] in individual studies that were included in the review? |  |
| Q10 | Did the review authors report on the sources of funding for the studies included in the review? |  |
| Q11 | If meta-analysis was performed did the review authors use appropriate methods for statistical combination of results? |  |
| Q12 | If meta-analysis was performed, did the review authors assess the potential impact of RoB in individual studies on the results of the meta-analysis or other evidence synthesis? |  |
| Q13 | Did the review authors account for RoB in individual studies when interpreting/discussing the results of the review? |  |
| Q14 | Did the review authors provide a satisfactory explanation for, and discussion of, any heterogeneity observed in the results of the review? |  |
| Q15 | If they performed quantitative synthesis did the review authors carry out an adequate investigation of publication bias [small study bias] and discuss its likely impact on the results of the review? |  |
| Q16 | Did the review authors report any potential sources of conflict of interest, including any funding they received for conducting the review? |  |

References

1. Mohammadi, S., Ghaderi, S. & Fatehi, F. Quantitative Susceptibility Mapping Values Quantification in Deep Gray Matter Structures for Relapsing-Remitting Multiple Sclerosis: A Systematic Review and Meta-Analysis. *Brain and Behavior* **14**, e70093 (2024).

2. Mohammadi, S., Ghaderi, S. & Fatehi, F. Putamen iron quantification in diseases with neurodegeneration: a meta-analysis of the quantitative susceptibility mapping technique. *Brain Imaging Behav* **18**, 1239–1255 (2024).

3. Voon, C. C., Wiltgen, T., Wiestler, B., Schlaeger, S. & Mühlau, M. Quantitative susceptibility mapping in multiple sclerosis: A systematic review and meta-analysis. *Neuroimage Clin* **42**, 103598 (2024).

4. De Lury, A. D. *et al.* Magnetic resonance imaging detection of deep gray matter iron deposition in multiple sclerosis: A systematic review. *J Neurol Sci* **453**, 120816 (2023).

5. Reeves, J. A. *et al.* Reliability of paramagnetic rim lesion classification on quantitative susceptibility mapping (QSM) in people with multiple sclerosis: Single-site experience and systematic review. *Mult Scler Relat Disord* **79**, 104968 (2023).

6. Verma, S., Goel, T. & Tanveer, M. Quantitative Susceptibility Mapping in Cognitive Decline: A Review of Technical Aspects and Applications. *Cogn Comput* **16**, 1992–2008 (2024).

7. Gupta, A. *et al.* The Use of Noncontrast Quantitative MRI to Detect Gadolinium-Enhancing Multiple Sclerosis Brain Lesions: A Systematic Review and Meta-Analysis. *AJNR Am J Neuroradiol* **38**, 1317–1322 (2017).

1. SMDs are usually estimated by Cohen's d or Hedges' g. Cohen's d divides the difference between sample means of a continuous response by the pooled standard deviation, but is subject to nonnegligible bias for small sample sizes. Hedges' g removes this bias with a correction factor. [↑](#footnote-ref-1)
